# Biomarkers of ocular manifestation in newly diagnosed giant cell arteritis

**DOI:** 10.1186/s12886-025-03997-x

**Published:** 2025-04-14

**Authors:** Jan Henrik Terheyden, Simon M. Petzinna, Lara C. Burg, Leon von der Emde, Charlotte Behning, Julie Jungblut, Katharina Reinking, Frank G. Holz, Thomas Ach, Maximilian W. M. Wintergerst, Valentin S. Schäfer, Robert P. Finger

**Affiliations:** 1 Department of Ophthalmology, University Hospital of Bonn, Venusberg-Campus 1, 53127 Bonn, Germany; 2 Department of Rheumatology and Clinical Immunology, Clinic of Internal Medicine III, University Hospital of Bonn, Bonn, Germany; 3 Institute for Medical Biometry, Informatics and Epidemiology, University of Bonn, Bonn, Germany; 4 Department of Ophthalmology, University Hospital Mannheim, University of Heidelberg, Mannheim, Germany

**Keywords:** Giant cell arteritis, Imaging, Ocular biomarker, Optical coherence tomography, Optical coherence tomography angiography

## Abstract

**Background:**

Giant cell arteritis (GCA) is a vasculitis of large and medium-sized vessels that causes severe ophthalmic complications. Timely diagnosis and disease monitoring may prevent permanent vision loss but biomarkers for early ocular involvement are scarce. This study evaluates early optical coherence tomography (OCT) and OCT angiography (OCTA) biomarkers of ocular involvement in newly diagnosed GCA.

**Methods:**

Newly diagnosed GCA patients and similarly aged controls were enrolled. Participants underwent ocular examination, including OCT and OCTA imaging of the macula and optic disc. OCT metrics included macular ganglion cell layer (GCL) and peripapillary retinal nerve fiber layer (pRNFL) thickness. OCTA parameters included vessel density (VD), vessel skeleton density, and vessel diameter index (VDI). Associations between imaging biomarkers and GCA symptoms (ordinal GCA symptom score) were analyzed using age-adjusted regression models.

**Results:**

We recruited 23 newly diagnosed GCA patients and 27 controls. VD and VDI in the deep retinal capillaries were significantly higher in GCA compared to controls (*p* ≤ 0.027). In patients reporting ocular symptoms, GCL thickness, volume and pRNFL thickness were increased in 11%, 22% and 17% of Early Treatment Diabetic Retinopathy Study subfields compared to controls (*p* ≤ 0.04). Additionally, GCL and pRNFL thicknesses were associated with GCA symptoms (*p* ≤ 0.041).

**Conclusions:**

OCT and OCTA imaging revealed structural and perfusion alterations in newly diagnosed GCA patients. Retinal microcirculation was altered, even regardless of the presence of ophthalmic symptoms. Structural changes correlated with systemic manifestations of GCA, suggesting a link between extracranial and intracranial involvement. Our findings underscore the potential diagnostic value of OCT and OCTA biomarkers for ocular involvement in GCA.

**Supplementary Information:**

The online version contains supplementary material available at 10.1186/s12886-025-03997-x.

## Introduction

Giant cell arteritis (GCA) is the most prevalent systemic vasculitis in the western world, primarily affecting large- and medium-sized vessels in individuals over 50 years of age [1]. The disease is characterized by vascular inflammation and remodeling, which can lead to stenosis and occlusion [2]. Notably, up to 70% of those affected by GCA experience ophthalmic manifestations [3], with permanent visual impairment reported in up to 37% of cases [3, 4]. Irreversible eye-related complications of GCA, arteritic anterior ischemic optic neuropathy (AION) and retinal artery occlusion, have a poor visual prognosis and may be refractory to glucocorticoid treatment [5–7].

Therefore, prompt diagnosis and immediate initiation of therapy are essential to reducing the risk of further ischemic complications [8]. Delayed or inaccurate diagnosis, which postpones high-dose systemic corticosteroid therapy, significantly increases the likelihood of permanent visual loss and contralateral eye involvement [9]. The implementation of Fast-Track Clinics, designed to expedite and optimize diagnostics for GCA, including ultrasound of the temporal and axillary arteries to evaluate macrovascular changes, has markedly decreased the incidence of permanent blindness in newly diagnosed GCA patients from 19 to 37% to 2–13% [4, 10–12]. Additionally, transorbital ultrasound has demonstrated potential in assessing central retinal flow velocity and structural ocular changes in GCA, underscoring the growing role of ocular imaging modalities in evaluating GCA-related complications [13].

However, while vascular and transorbital ultrasound are effective in detecting GCA-associated macrovascular changes, they lack the ability to assess microvascular alterations in ocular anatomy and perfusion induced by GCA. In this regard, optical coherence tomography (OCT) and OCT angiography (OCTA) may provide high-resolution imaging capable of evaluating subtle ocular changes, making them promising tools for the early detection of ophthalmic manifestations of GCA [14]. Most existing studies on OCT and OCTA biomarkers in GCA have focused on patients with advanced or established ophthalmologic manifestation [15, 16], leaving a significant gap in identifying early indicators of ocular involvement. This study aims to address this gap by investigating the diagnostic potential of OCT and OCTA as early imaging biomarkers in newly diagnosed GCA patients compared to healthy controls.

## Methods

### Patient characteristics

Patients with newly diagnosed, untreated GCA were enrolled at the Department of Ophthalmology and the Department of Rheumatology at the University Hospital Bonn, Germany, between October 1, 2018, and May 31, 2022. Previous findings in the same cohort have been published independently of the present study [30] and focused on associations between ocular and non-ocular imaging biomarkers of GCA. In contrast to the goal of our previous study, the aim of this study was to investigate changes in ocular biomarkers in newly diagnosed GCA patients compared to control participants. Diagnoses of GCA were made by a board-certified rheumatologist. Eligible participants were required to be over 50 years of age. Patients were required to exhibit elevated serum inflammatory markers (C-reactive protein [CRP] > 10 mg/L), and have a history of glucocorticoid use not exceeding seven days prior to enrollment, in addition to meeting the ACR-EULAR classification criteria [17]. Patients not meeting these criteria were excluded. Healthy control participants of a similar age group were recruited through the Department of Ophthalmology at the University Hospital Bonn. Further exclusion criteria included concurrent rheumatologic diseases other than GCA and polymyalgia rheumatica, high myopia, ischemic retinopathies, and relevant media opacities impairing the interpretation of ocular imaging. Additionally, eyes with a history or evidence of AION, retinal artery occlusions, or imaging of insufficient quality (OCT signal strength < 20 dB or OCTA index < 7) were excluded, while participants were allowed to have self-reported ocular symptoms.

### Clinical assessment

At study inclusion, demographic data, glucocorticoid treatment history, cardiovascular risk factors, and the history of neurological and ophthalmological diseases were recorded for each participant. We conducted a single examination of all participants’ eyes, which included assessing best-corrected visual acuity (BCVA), intraocular pressure and a clinical evaluation as well as OCT and OCTA imaging of the macula and the optic disc. Additionally, participants with GCA underwent a comprehensive physical examination conducted by a board-certified rheumatologist, as well as laboratory assessments, including CRP, hemoglobin, leukocyte count, and platelet count. Ultrasound examinations of the superficial temporal arteries and their branches, as well as the facial, axillary, carotid, and vertebral arteries, were performed as described before [13, 18, 19]. These ultrasound assessments were conducted by board-certified rheumatologists with extensive experience in GCA-related ultrasound imaging (> 1000 examinations) or by supervised medical students under their guidance.

### Ocular imaging

The same protocol and devices were used across all participants (spectral-domain OCT: Spectralis HRA + OCT, Heidelberg Engineering, Heidelberg, Germany; swept-source OCTA: PLEX Elite 9000, Carl Zeiss Meditec, Dublin, California). The OCT imaging protocol included a volume scan of the macula (20°×15°, 19 single horizontal B-Scans, 25 frames, including automatic real-time tracking), centered on the fovea and peripapillary ring scans (diameter 3.5 mm, 100 frames, including automatic real-time tracking), centered on the optic disc. The OCTA imaging protocol included 6 × 6 mm cube scans centered at the macula and the optic disc, respectively, at a frequency of 100,000 A-scans per second. We performed imaging without pupil dilation, scheduling the eye exam within the first week after initial diagnosis of GCA. Both OCT and OCTA scans were automatically segmented using the proprietary algorithms implemented in the respective devices, and reviewed by two readers each (JHT and JJ) to allow for manual corrections of the segmentations where necessary. From OCT data, a macular ganglion cell layer (GCL) thickness map and a peripapillary retinal nerve fiber layer (pRNFL) thickness map were automatically calculated in the Heidelberg Eye Explorer (HEYEX 2, Heidelberg Engineering, Heidelberg, Germany). From OCTA data, en face images were generated as previously reported [20]. In brief, the proprietary algorithm calculated separate flow maps of the superficial retinal layer (flow information from retinal nerve fiber layer to inner plexiform layer), deep retinal layer (inner nuclear layer to Henle fiber layer) and choriocapillaris layer. Within the software, projections of the superficial vessels were removed from the en face images of the deep retinal layer and choriocapillaris layer.

### Image data extraction

Subfield GCL and pRNFL thickness data were extracted from the OCT thickness maps. The subfields of the macular volume scan followed the Early Treatment Diabetic Retinopathy Study grid [ETDRS grid]. OCTA en face-maps were first processed with Fiji (based on ImageJ, version 1.51w) in order to obtain the perfusion variables for further analysis. We binarized the en face image showing the perfusion of the superficial and deep retinal plexus, using an automated algorithm [20, 21]. The parameters vessel density, vessel skeleton density and vessel diameter index were calculated for use as outcome variables [22]. Vessel densitiy represents a global marker of the retinal perfusion while vessel skeleton density and vessel diameter index are more affected by longitudinal and diametral changes in the vessel perfusion, respectively. The en face image of the choriocapillaris perfusion was binarized, using an automated technique that retains flow voids exceeding the intercapillary distance [23]. We extracted the global proportion of flow deficits per en face image, as well as their number and average size.

### Statistical analysis

From each participant, one study eye was selected for study inclusion, following a consistent algorithm across participants. Right eyes were preferred if both eyes were eligible as study eyes. In participants with GCA, eyes with a history of reporting ocular symptoms of GCA were preferred in the selection process. We analyzed the data using R, version 4.3.0 (R Core Team, Vienna, Austria). OCT and OCTA parameters between both groups were compared using linear regression models that included the OCT and OCTA variables as dependent variables, and the group membership (GCA or control participant) as independent variables, controlling for participants’ age. We also compared participants with a history of reporting ocular symptoms of GCA to controls in similar linear regression analyses (dependent variables: OCTA and OCTA biomarkers, independent variables: Ocular symptom score, age). To obtain the symptom score, we added 1 point for each GCA symptom (night sweat, unintentional weight loss, fatigue, coughing, headaches, jaw pain, jaw claudication, tongue burning, temporal touch sensitivity, visual symptoms), thus obtaining values between 0 and 10. All regression analyses were preceded by confirmation of normal distribution of residuals. We did not adjust the analyses for multiple comparisons due to the exploratory nature of our study. P-values < 0.05 were considered statistically significant.

### Ethical approval

The study was conducted in accordance with the Declaration of Helsinki and received approval from the ethics committee of the University Hospital Bonn, Germany (reference number: 097/18). Written informed consent was obtained from every patient prior to inclusion in the study.

## Results

### Patient characteristics

We included 50 participants, 23 patients with GCA and 27 controls. Among GCA patients, 14 individuals (60.9%) had concurrent polymyalgia rheumatica, while no polymyalgia rheumatica was present in the remaining 9 participants. On average, GCA participants exhibited a mean CRP level of 67.3 ± 40.2 mg/l. In vascular ultrasound, a mean of 8.7 ± 2.8 vessels were classified pathological. The average GCA symptom score, as defined above, was 4.0 ± 3.2. Seven GCA participants (30.4%) reported ocular symptoms that lacked structural ophthalmic manifestations of GCA such as AION. These individuals were included in a subgroup analysis described below.

Comparing GCA participants and healthy controls, gender distribution was balanced, with 52% female and 48% male participants in both groups. However, GCA participants were, on average, older than controls (Table 1); therefore, all analyses were adjusted for age. The prevalence of relevant systemic conditions was qualitatively comparable between groups (Table 1).

**Table 1:** Participant characteristics

	GCA (*n* = 23)	Controls (*n* = 27)
Age [years]	75.3 ± 8.8	68.1 ± 6.0
Sex Female (%) Male (%)	12 (52.2%) 11 (47.8%)	14 (51.9%) 13 (48.1%)
Medical history:		
Obesity (%)	2 (8.7)	9 (33.3)
Hypertension (%)	14 (60.9)	10 (37.0)
Diabetes (%)	3 (13)	0 (0)
Atherosclerosis (%)	7 (30.4)	15 (55.6)
Neurodegenerative conditions (%)	0 (0)	0 (0)
BCVA (study eye) [logMAR units]	0.83 ± 0.25	0.88 ± 0.20
Foveal GCL volume [mm³]	0.02 ± 0.01	0.01 ± 0.01
Global pRNFL thickness [µm]	94.5 ± 17.0	92.7 ± 6.3
Superficial retinal plexus macular vessel density	0.24 ± 0.05	0.24 ± 0.04
Deep retinal plexus macular vessel density	0.16 ± 0.02	0.15 ± 0.02

Parameters are presented as frequencies n (percentage) or mean ± standard deviation. Abbrv.: BCVA = best-corrected visual acuity; GCA = giant cell arteritis, GCL = ganglion cell layer, pRNFL = peripapillary retinal nerve fibre Layer

**Fig. 1:**
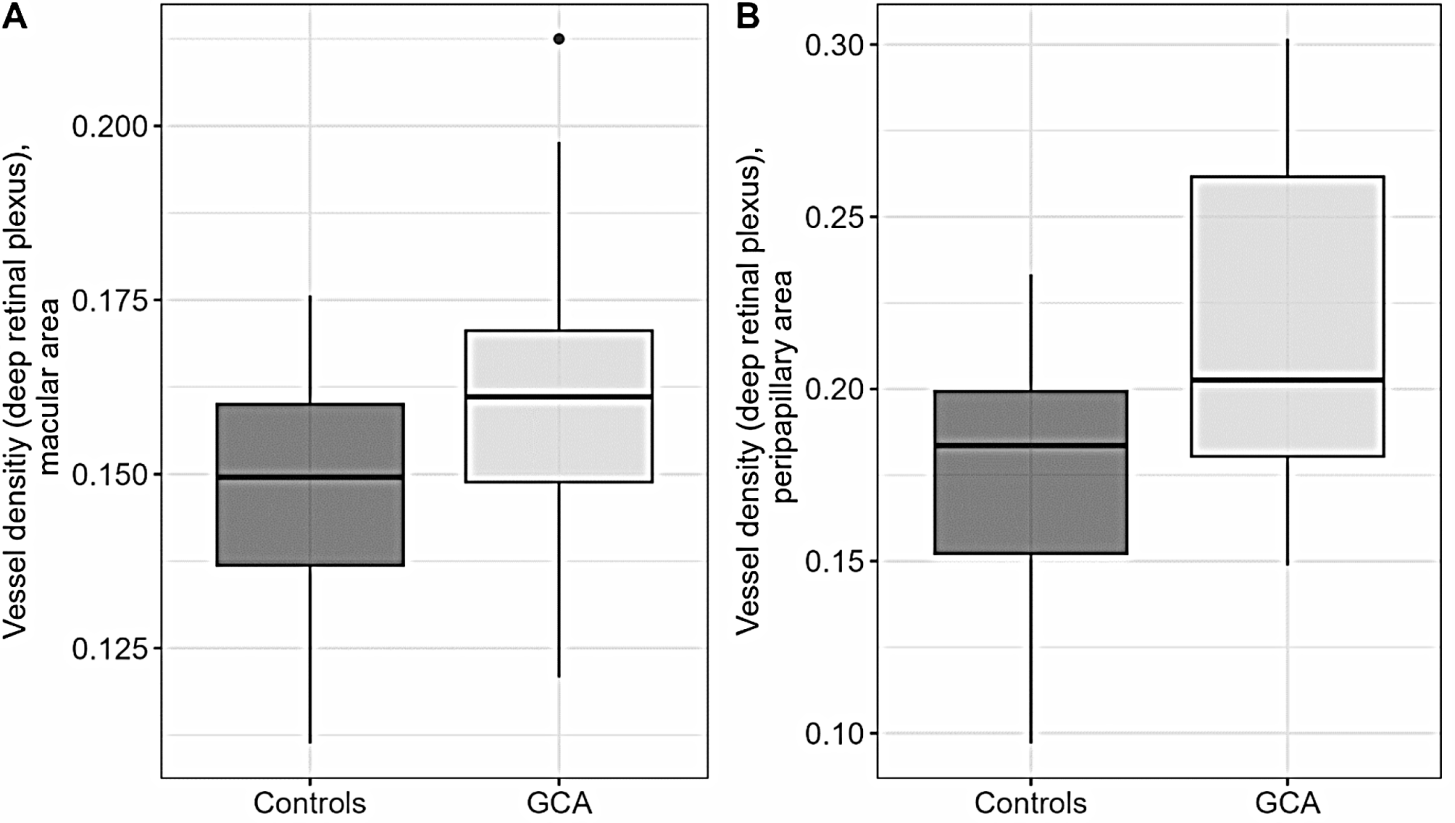
Vessel Densities on Optical Coherence Tomography Angiography of the Macula and Optic Nerve. GCA = giant cell arteritis

The vessel density of the deep retinal capillaries on OCTA was significantly higher in individuals with GCA compared to controls, while controlling the analysis for age. This effect was present both in analyses of OCTA scans centered at the macula and at the optic nerve (macula: β = 0.01, *p* = 0.027; peripapillary: β = 0.04, *p* = 0.004; Fig. 1). Similarly, the vessel diameter index was significantly higher in participants with GCA compared to controls across these scan locations (macula: β = 69398, *p* = 0.017; peripapillary: β = 309862, *p* = 0.001). Vessel parameters of the superficial capillary plexus and the number and average size of choriocapillaris flow deficits were not different between the groups (*p* ≥ 0.228). On OCT, subfield macular GCL volumes and thicknesses were not significantly different between GCA and control participants of our study (*p* ≥ 0.133 and 0.168, respectively). Also, pRNFL thickness was not significantly different between the groups (*p* ≥ 0.160).

Figure 1 illustrates vessel densities measured using optical coherence tomography angiography in patients with giant cell arteritis and controls. Panel (a) shows the vessel densities of the macula, while panel (b) displays the vessel densities of the optic nerve head.

In a subgroup analysis, we compared the seven study participants with GCA and self-reported ocular symptoms to control participants. Of note, none of the participants in this analysis had an ophthalmic complication of GCA such as AION or retinal artery occlusion. Despite this, macular GCL volume and thickness in the outer superior subfield as well as the foveal GCL volume were significantly higher in participants with GCA compared to controls (β = 0.04, *p* = 0.020; β = 7.61, *p* = 0.016; and β = 0.01, *p* = 0.017, respectively). Also, pRNFL thickness was significantly increased in GCA participants globally and in the temporal inferior subfield (β = 16.88, *p* = 0.001 and β = 29.76, *p* = 0.04, respectively). Similarly to the overall results, vessel perfusion density and vessel diameter index of the deep retinal plexus on OCTA were significantly higher in participants with GCA (macular scans: β = 0.02, *p* = 0.021; β = 85044, *p* = 0.016; optic nerve scans: β = 0.04, *p* = 0.044; β = 314979, *p* = 0.005).

Considering all 23 participants with GCA, OCT biomarkers were significantly associated with a higher number of GCA symptoms in the symptom score. Specifically, we found the symptom score to be associated with macular GCL thickness in the inner and outer superior subfields as well as the outer temporal subfields (β = 1.85, *p* = 0.024; β = 1.57, *p* = 0.018; β = 0.78, *p* = 0.043), with macular GCL volume in the outer nasal, superior and temporal subfields (β = 0.01, *p* = 0.041; β = 0.01, *p* = 0.023; β < 0.01, *p* = 0.035, respectively), and with pRNFL thicknesses globally as well as in the temporal inferior and nasal superior subfields (β = 3.87, *p* = 0.007; β = 5.36, *p* = 0.033; β = 6.67, *p* = 0.011) (Table 2). In contrast to this, none of the OCTA parameters showed significant associations with the GCA symptom score (*p* ≥ 0.093).

**Table 2:** Association between peripapillary retinal nerve fiber layer thickness as well as macular ganglion cell layer thickness and giant cell arteritis symptoms score (sum score of the presence of night sweat, unintentional weight loss, fatigue, coughing, headaches, jaw pain, jaw claudication, tongue burning, temporal touch sensitivity and visual symptoms) in the subset of 23 affected participants, controlled for age

	β [95% CI]	*p*-value
Global pRNFL thickness	3.87 [1.23; 6.51]	0.007
Subfield pRNFL thickness		
Temporal inferior Temporal Temporal superior Nasal superior Nasal Nasal inferior	5.36 [0.50; 10.22] 3.04 [-0.44; 6.53] 0.97 [-3.34; 5.28] 6.67 [1.81; 11.54] 3.56 [-0.02; 7.14] 4.70 [-2.71; 12.11]	0.033 0.082 0.637 0.011 0.051 0.195
Subfield macular GCL thickness		
Inner inferior Inner nasal Inner superior Inner temporal Outer inferior Outer nasal Outer superior Outer temporal	1.10 [-0.15; 2.35] 1.19 [-0.39; 2.76] 1.85 [0.27; 3.43] 1.05 [-0.73; 2.83] 0.46 [-0.49; 1.40] 0.84 [-0.06; 1.74] 1.57 [0.31; 2.84] 0.78 [0.03; 1.54]	0.080 0.131 0.024 0.232 0.322 0.065 0.018 0.043
Subfield macular GCL volume		
Inner inferior Inner nasal Inner superior Inner temporal Outer inferior Outer nasal Outer superior Outer temporal	< 0.01 [<-0.01; <0.01] < 0.01 [<-0.01; 0.01] < 0.01 [<-0.01; <0.01] < 0.01 [<-0.01; <0.01] < 0.01 [<-0.01; 0.01] < 0.01 [< 0.01; 0.01] < 0.01 [< 0.01; 0.01] < 0.01 [< 0.01; 0.01]	0.128 0.208 0.056 0.471 0.380 0.041 0.023 0.035

CI, confidence interval; GCL, ganglion cell layer; pRNFL, peripapillary retinal nerve fiber layer

Analyses are controlled for age to account for its potential confounding effect

## Discussion

The results of our study highlight the potential utility of OCT and OCTA as imaging-based diagnostic biomarkers in newly diagnosed GCA patients. We found a pronounced alteration of the outer retinal microperfusion in individuals with GCA, even in the absence of ophthalmic complications. In addition, the structure of the ganglion cell layer also exhibited changes in GCA patients with ocular symptoms, highlighting the need to better understand pre-clinical stages of ocular involvement in GCA. Further, our study suggests that structural retinal changes in GCA are associated with the severity of GCA symptoms. Therefore, OCT and OCTA imaging biomarkers may be of diagnostic, monitoring, and prognostic value and therefore help preventing manifest ophthalmic complications of GCA.

Our main finding at least partly contrasts a previous study investigating OCTA biomarkers in 16 individuals without ocular complications, which reported retinal microperfusion changes limited to the peripapillary region but not observed in macular scans [24]. Moreover, our cohort exhibited increased vessel density in GCA patients compared to controls, differing from Vannozzi et al. findings where vessel density was reduced. An increase in capillary density was also reported in fellow eyes with arteritic AION in a published case series [16]. These conflicting results may reflect distinct stages of ocular involvement in GCA and compensatory microcirculatory mechanisms, such as myogenic compensation or the Ostroumov-Bayliss effect, which requires further evaluation in larger scale and longitudinal studies [25].

The above-mentioned study also reported reduced inferior subfield pRNFL thickness in GCA patients without ocular involvement compared to controls, a finding not replicated in our cohort. However, it remains unclear whether this discrepancy could be attributed to age differences, which only our study accounted for. Considering potential additional novel vessel biomarker on structural OCT images, a study investigating retinal vessel reflectivity in a mixed cohort of GCA patients with and without ocular complications found alterations in retinal vessel reflectivity to be altered in participants with ocular complications of GCA, but not in patients without GCA-related eye complications [15]. In summary, the results of our study and published results of OCT and OCTA imaging in new-onset GCA highlight the potential of ophthalmic imaging modalities in aiding the diagnosis and risk stratification in GCA patients.

Symptomatic eyes without GCA complications were structurally altered and had retinal microperfusion changes across the posterior pole in our cohort. This finding suggests that imaging biomarkers could be associated with early ocular involvement in GCA, since structural thickening is a possible result of ischemia [26]. In this context, the temporal relationship between perfusion changes and structural changes in the inner retina remain to be addressed in further detail. On a pathophysiological basis, AION is caused by occlusion of the short posterior ciliary arteries supplying the optic nerve head, leading to ischemia in the optic nerve’s laminar and prelaminar regions, sectoral retinal opacification, and optic disc edema. These pathological changes result in thickening of the inner retinal nerve fiber and ganglion cell layers, followed by irreversible loss of inner retinal structure [27–29]. The pronounced OCTA and structural OCT biomarker changes in symptomatic eyes without AION are in line with our published findings on ocular ultrasound in GCA patients, where the peak systolic flow in the central retinal artery was significantly different in symptomatic eyes of GCA patients from controls [13, 30]. Up to 70% of GCA patients report visual symptoms [31] but the prognostic value of these symptoms remain largely unknown and unspecific. Thus, our study adds to the literature that OCT and OCTA may provide early biomarkers of ocular involvement in GCA for the prevention of complications causing visual impairment in patients with GCA.

Comparing systemic and ophthalmological manifestations of GCA, structural thickening of the GCL and pRNFL correlated with the number of general GCA symptoms. This aligns with recent research indicating an association between extracranial and intracranial manifestations of GCA [13]. Therefore, our study highlights the potential of OCT and OCTA in disease monitoring and as endpoints in clinical trials. In this context, ophthalmic imaging modalities offer the unique advantage of assessing tissues perfused by different branches of the ophthalmic artery, such as the long ciliary and central retinal arteries [31]. Future studies incorporating macro- and microcirculatory imaging should explore the interplay between these vascular pathways and their temporal changes.

The strengths of our study include the rigorous examination of study participants which allowed us to exclude relevant comorbidities affecting ophthalmic imaging, the use of a highly reproducible diagnostic algorithm to confirm the presence of GCA, the availability of a broad spectrum of clinical data (including patient-reported data on visual symptoms). Our study adds to previous findings in the same cohort [30], which suggested that OCTA parameters of the retinal vasculature and ultrasound parameters of the central retinal artery are complementary assessments that cannot replace one another. Our study is limited by its exploratory nature due to which we did not correct our analyses for multiple testing and the presence of potential confounders beyond age (e.g. presence of diabetes and hypertension). However, the main results in the overall cohort were reproducible between scans located at the fovea and the optic disc. Another limitation is the sample size of our study. Nonetheless, our analyses add considerable value to the literature given the few published results on ocular imaging in GCA. Finally, the distributions between the GCA and control cohorts were not perfectly balanced and the GCA group was older than the control group. We thus controlled for age in the primary endpoint analyses of our study. Furthermore, statistical testing suggested that obesity was more prevalent in the GCA cohort, which may have impacted the results of our study, while none of the observed differences in the prevalence of other conditions differed significantly between the groups (Supplementary Table 1). The lack of longitudinal follow-up precludes conclusions about the risk of symptomatic GCA patients developing AION or arterial occlusions, which should be addressed in future studies.

In conclusion, our study underscores the potential of OCTA biomarkers (deep retinal layer VD and VDI) as well as structural OCT biomarkers (GCL and pRNFL) for diagnosing ocular involvement in GCA. Pending further independent evaluation of the biomarkers’ predictive power, ophthalmic imaging could become useful for guiding personalized treatments to reduce the visual burden of GCA.

## Electronic supplementary material

Below is the link to the electronic supplementary material.

Supplementary Material 1

## Data Availability

Data supporting the manuscript are not publicly available to retain anonymity of the research participants but are available on reasonable request from the authors.

## References

[1] Buttgereit F, Dejaco C, Matteson EL, Dasgupta B. Polymyalgia rheumatica and giant cell arteritis: A systematic review. JAMA. 2016;315:2442–58. 10.1001/jama.2016.5444. 27299619 10.1001/jama.2016.5444

[2] Hoffman GS. Giant cell arteritis. Ann Intern Med. 2016;165:ITC65–80. 10.7326/AITC201611010. 27802475 10.7326/AITC201611010

[3] Salvarani C, Cimino L, Macchioni P, Consonni D, Cantini F, Bajocchi G, et al. Risk factors for visual loss in an Italian population-based cohort of patients with giant cell arteritis. Arthritis Rheum. 2005;53:293–7. 10.1002/art.21075. 15818722 10.1002/art.21075

[4] Patil P, Williams M, Maw WW, Achilleos K, Elsideeg S, Dejaco C, et al. Fast track pathway reduces sight loss in giant cell arteritis: results of a longitudinal observational cohort study. Clin Exp Rheumatol. 2015;33:S–103. 26016758

[5] Danesh-Meyer H, Savino PJ, Gamble GG. Poor prognosis of visual outcome after visual loss from giant cell arteritis. Ophthalmology. 2005;112:1098–103. 10.1016/j.ophtha.2005.01.036. 15885780 10.1016/j.ophtha.2005.01.036

[6] Liu GT, Glaser JS, Schatz NJ, Smith JL. Visual morbidity in giant cell arteritis. Clinical characteristics and prognosis for vision. Ophthalmology. 1994;101:1779–85. 10.1016/s0161-6420(94)31102-x. 7800356 10.1016/s0161-6420(94)31102-x

[7] Hayreh SS, Podhajsky PA, Zimmerman B. Ocular manifestations of giant cell arteritis. Am J Ophthalmol. 1998;125:509–20. 10.1016/s0002-9394(99)80192-5. 9559737 10.1016/s0002-9394(99)80192-5

[8] Schäfer VS, Petzinna SM, Schmidt WA. Neues in der bildgebung von Großgefäßvaskulitiden. [News on the imaging of large vessel vasculitis]. Z Rheumatol. 2024;83:800–11. 10.1007/s00393-024-01565-0. 39271483 10.1007/s00393-024-01565-0

[9] Dejaco C, Brouwer E, Mason JC, Buttgereit F, Matteson EL, Dasgupta B. Giant cell arteritis and polymyalgia rheumatica: current challenges and opportunities. Nat Rev Rheumatol. 2017;13:578–92. 10.1038/nrrheum.2017.142. 28905861 10.1038/nrrheum.2017.142

[10] Diamantopoulos AP, Haugeberg G, Lindland A, Myklebust G. The fast-track ultrasound clinic for early diagnosis of giant cell arteritis significantly reduces permanent visual impairment: towards a more effective strategy to improve clinical outcome in giant cell arteritis? Rheumatology (Oxford). 2016;55:66–70. 10.1093/rheumatology/kev289. 26286743 10.1093/rheumatology/kev289

[11] Monti S, Bartoletti A, Bellis E, Delvino P, Montecucco C. Fast-Track ultrasound clinic for the diagnosis of giant cell arteritis changes the prognosis of the disease but not the risk of future relapse. Front Med (Lausanne). 2020;7:589794. 10.3389/fmed.2020.589794. 33364248 10.3389/fmed.2020.589794 PMC7753207

[12] Schmidt WA. Ultrasound in the diagnosis and management of giant cell arteritis. Rheumatology (Oxford). 2018;57:ii22–31. 10.1093/rheumatology/kex461. 29982780 10.1093/rheumatology/kex461

[13] Petzinna SM, Burg LC, Bauer C-J, Karakostas P, Terheyden JH, Behning C, et al. Transorbital ultrasound in the diagnosis of giant cell arteritis. Rheumatology (Oxford). 2024;63:2379–86. 10.1093/rheumatology/keae287. 38759118 10.1093/rheumatology/keae287

[14] Aumann S, Donner S, Fischer J, Müller F. High resolution imaging in microscopy and ophthalmology: new frontiers in biomedical optics: optical coherence tomography (OCT): principle and technical realization. Cham (CH); 2019. 32091846

[15] Klefter ON, Hansen MS, Willerslev A, Faber C, Terslev L, Jensen MR, et al. Optical coherence tomography of peripapillary vessels in giant cell arteritis and ischaemic ocular disease. Neuroophthalmology. 2022;46:383–9. 10.1080/01658107.2022.2113901. 36544584 10.1080/01658107.2022.2113901 PMC9762795

[16] Gaier ED, Gilbert AL, Cestari DM, Miller JB. Optical coherence tomographic angiography identifies peripapillary microvascular dilation and focal non-perfusion in giant cell arteritis. Br J Ophthalmol. 2018;102:1141–6. 10.1136/bjophthalmol-2017-310718. 29122818 10.1136/bjophthalmol-2017-310718

[17] Ponte C, Grayson PC, Robson JC, Suppiah R, Gribbons KB, Judge A, et al. 2022 American college of rheumatology/eular classification criteria for giant cell arteritis. Ann Rheum Dis. 2022;81:1647–53. 10.1136/ard-2022-223480. 36351706 10.1136/ard-2022-223480

[18] Schäfer VS, Chrysidis S, Schmidt WA, Duftner C, Iagnocco A, Bruyn GA, et al. OMERACT definition and reliability assessment of chronic ultrasound lesions of the axillary artery in giant cell arteritis. Semin Arthritis Rheum. 2021;51:951–6. 10.1016/j.semarthrit.2021.04.014. 34140184 10.1016/j.semarthrit.2021.04.014

[19] Schäfer VS, Juche A, Ramiro S, Krause A, Schmidt WA. Ultrasound cut-off values for intima-media thickness of Temporal, facial and axillary arteries in giant cell arteritis. Rheumatology (Oxford). 2017;56:1632. 10.1093/rheumatology/kex289. 28859330 10.1093/rheumatology/kex289

[20] Terheyden JH, Wintergerst MWM, Falahat P, Berger M, Holz FG, Finger RP. Automated thresholding algorithms outperform manual thresholding in macular optical coherence tomography angiography image analysis. PLoS ONE. 2020;15:e0230260. 10.1371/journal.pone.0230260. 32196538 10.1371/journal.pone.0230260 PMC7083322

[21] Otsu N. A threshold selection method from Gray-Level histograms. IEEE Trans Syst Man Cybern. 1979;9:62–6. 10.1109/TSMC.1979.4310076.

[22] Kim AY, Chu Z, Shahidzadeh A, Wang RK, Puliafito CA, Kashani AH. Quantifying microvascular density and morphology in diabetic retinopathy using Spectral-Domain optical coherence tomography angiography. Invest Ophthalmol Vis Sci. 2016;57:OCT362–70. 10.1167/iovs.15-18904. 27409494 10.1167/iovs.15-18904 PMC4968771

[23] Zhang Q, Shi Y, Zhou H, Gregori G, Chu Z, Zheng F, et al. Accurate Estimation of choriocapillaris flow deficits beyond normal intercapillary spacing with swept source OCT angiography. Quant Imaging Med Surg. 2018;8:658–66. 10.21037/qims.2018.08.10. 30211033 10.21037/qims.2018.08.10 PMC6127524

[24] Vannozzi L, Nicolosi C, Vicini G, Bacherini D, Giattini D, Urban ML, et al. Optical coherence tomography angiography findings in patients affected by giant cell arteritis, with and without ocular involvement: a pilot study. Front Med (Lausanne). 2024;11:1408821. 10.3389/fmed.2024.1408821. 39188882 10.3389/fmed.2024.1408821 PMC11345371

[25] Bayliss WM. On the local reactions of the arterial wall to changes in internal pressure. J Physiol. 1902;28:220–31. 16992618 10.1113/jphysiol.1902.sp000911 PMC1540533

[26] Mendoza-Santiesteban CE, Patel N, Monaco C, Hedges TR. Distinctive pattern of ganglion cell layer loss in early ischemic optic neuropathy. Invest Ophthalmol Vis Sci. 2014:3391.

[27] Biousse V, Newman NJ. Ischemic optic neuropathies. N Engl J Med. 2015;372:2428–36. 10.1056/NEJMra1413352. 26083207 10.1056/NEJMra1413352

[28] Chen Q, Chen W, Feng C, Gong D, Zhang J, Bi Y, et al. Giant cell arteritis presenting with ocular symptoms: clinical characteristics and multimodal imaging in a Chinese case series. Front Med (Lausanne). 2022;9:885463. 10.3389/fmed.2022.885463. 35795624 10.3389/fmed.2022.885463 PMC9251180

[29] Singh AG, Kermani TA, Crowson CS, Weyand CM, Matteson EL, Warrington KJ. Visual manifestations in giant cell arteritis: trend over 5 decades in a population-based cohort. J Rheumatol. 2015;42:309–15. 10.3899/jrheum.140188. 25512481 10.3899/jrheum.140188 PMC4367485

[30] Petzinna SM, Terheyden JH, Burg LC, Bauer C-J, Karakostas P, Behning C et al. Imaging of ophthalmic manifestations: optical coherence tomography angiography and transorbital ultrasound in giant cell arteritis. Rheumatol Int. 2025. 10.1007/s00296-025-05800-y PMC11814037 39932562

[31] Vodopivec I, Rizzo JF. Ophthalmic manifestations of giant cell arteritis. Rheumatology (Oxford). 2018;57:ii63–72. 10.1093/rheumatology/kex428. 29986083 10.1093/rheumatology/kex428

