# Supplementary material for: Biomarkers of ocular manifestation in newly diagnosed giant cell arteritis
Source: BMC Ophthalmol. 2025 Apr 14;25:201. doi: 10.1186/s12886-025-03997-x (PMC11995548; doi:10.1186/s12886-025-03997-x)
Supplement: Supplementary file 1 — Supplementary Material 1 [file 12886_2025_3997_MOESM1_ESM.docx]

**Biomarkers of Ocular Manifestation in Newly Diagnosed Giant Cell Arteritis**

**SUPPLEMENT**

Jan Henrik Terheyden^1†^, MD; Simon M. Petzinna^2†^, MD; Lara C. Burg, MD^2^; Leon von der Emde^1^, MD; Charlotte Behning^3^; Julie Jungblut^1^; Katharina Reinking^1^, MD; Frank G. Holz^1^, MD; Thomas Ach^1^, MD; Maximilian W. M. Wintergerst^1^, MD; Valentin S. Schäfer^2‡^, MD; Robert P. Finger^1‡^, MD, PhD

^1^ Department of Ophthalmology, University Hospital of Bonn, Bonn, Germany

^2^ Department of Rheumatology and Clinical Immunology, Clinic of Internal Medicine III, University Hospital of Bonn, Bonn, Germany

^3^ Institute for Medical Biometry, Informatics and Epidemiology, University of Bonn, Bonn, Germany

†authors contributed equally to this work

‡authors contributed equally to this work

| **Supplementary Table 1.** Participant characteristics. P-values were obtained as follows: For categorical data, Chi-Square tests with Yates’ continuity correction (as implemented in R version 4.3.0) were used or Fisher's exact test if the number of expected frequencies was lower than five. For metrical, Mann-Whitney test was used for asymmetrical data or a Welch t-test, if the data was asymptotically normal distributed. | | | | | | |
| --- | --- | --- | --- | --- | --- | --- |
|  | **GCA** (n=23) | N/A | **Controls** (n=27) | N/A | **p-value** |  |
| Age [years] | 75.3 ± 8.8 | - | 68.1 ± 6.0 | - | 0.002^a^ |  |
| Sex Female (%)  Male (%) | 12 (52.2%) 11 (47.8%) | - | 14 (51.9%) 13 (48.1%) | - | 1.000^b^ |  |
| Medical history: |  | - |  | - |  |  |
| Obesity (%) | 2 (8.7) | - | 9 (33.3) | - | 0.045^c^ |  |
| Hypertension (%) | 14 (60.9) | - | 10 (37.0) | - | 0.162^b^ |  |
| Diabetes (%) | 3 (13) | - | 0 (0) | - | 0.090^c^ |  |
| Atherosclerosis (%) | 7 (30.4) | - | 15 (55.6) |  | 0.134^b^ |  |
| Neurodegenerative conditions (%) | 0 (0) | - | 0 (0) | - | - |  |
| BCVA (study eye) [logMAR units] | 0.8317 ± 0.2487 | 5 | 0.8831 ± 0.1983 | 1 | 0.749^d^ |  |
| Foveal GCL volume [mm³] | 0.0181 ± 0.01078 | 2 | 0.01370 ± 0.0056 | - | 0.228^d^ |  |
| Global pRNFL thickness [µm] | 94.53 ± 16.99 | 6 | 92.74 ± 6.247 | 4 | 0.683^a^ |  |
| Superficial retinal plexus macular vessel density | 0.2403 ± 0.04455 | 3 | 0.2359 ± 0.04075 | - | 0.733^a^ |  |
| Deep retinal plexus macular vessel density | 0.1604 ± 0.02288 | 2 | 0.1478 ± 0.01555 | - | 0.038^a^ |  |
|  |  |  |  |  |  |  |

Parameters are presented as frequencies n (percentage) or mean ± standard deviation. Abbrv.: N/A = not available, BCVA = best-corrected visual acuity; GCA = giant cell arteritis, GCL = ganglion cell layer, pRNFL = peripapillary retinal nerve fibre Layer

Statistical test used: ^a^ Welch t-test, ^b^ Chi-Square test, ^c^ Fisher's exact test, ^d^ Mann-Whitney test
